# Bacteriophages are the major drivers of *Shigella flexneri* serotype 1c genome plasticity: a complete genome analysis

**DOI:** 10.1186/s12864-017-4109-4

**Published:** 2017-09-12

**Authors:** Pawan Parajuli, Marcin Adamski, Naresh K. Verma

**Affiliations:** 1 Division of Biomedical Science and Biochemistry, Research School of Biology, The Australian National University, ACT Canberra, Australia; 2 Computational Biology and Bioinformatics Unit, Research School of Biology, The Australian National University, ACT Canberra, Australia

**Keywords:** *Shigella flexneri*, Shigellosis, Bacteriophage, O-antigen

## Abstract

**Background:**

*Shigella flexneri* is the primary cause of bacillary dysentery in the developing countries. *S. flexneri* serotype 1c is a novel serotype, which is found to be endemic in many developing countries, but little is known about its genomic architecture and virulence signatures. We have sequenced for the first time, the complete genome of *S. flexneri* serotype 1c strain Y394, to provide insights into its diversity and evolution.

**Results:**

We generated a high-quality reference genome of *S. flexneri* serotype 1c using the hybrid methods of long-read single-molecule real-time (SMRT) sequencing technology and short-read MiSeq (Illumina) sequencing technology. The Y394 chromosome is 4.58 Mb in size and shares the basic genomic features with other *S. flexneri* complete genomes. However, it possesses unique and highly modified O-antigen structure comprising of three distinct O-antigen modifying gene clusters that potentially came from three different bacteriophages. It also possesses a large number of hypothetical unique genes compared to other *S. flexneri* genomes.

**Conclusions:**

Despite a high level of structural and functional similarities of Y394 genome with other *S. flexneri* genomes, there are marked differences in the pathogenic islands. The diversity in the pathogenic islands suggests that these bacterial pathogens are well adapted to respond to the selection pressures during their evolution, which might contribute to the differences in their virulence potential.

**Electronic supplementary material:**

The online version of this article (10.1186/s12864-017-4109-4) contains supplementary material, which is available to authorized users.

## Background

*Shigella* species are the Gram-negative bacteria that cause shigellosis – a leading cause of bacillary dysentery in developing countries. Annually, 125 million cases of endemic shigellosis occur in Asia alone and children under 5 years of age are at the highest risk of illness and death [1]. Among the four species of *Shigella*, *S. flexneri* is the primary cause of shigellosis in developing countries accounting up to 66% of all *Shigella* species infections [2]. There are 19 different *S. flexneri* serotypes based on their antigenic determinants present on the O-antigen of the outer membrane lipopolysaccharide (LPS) [3]. The O-antigen modification that results in serotype conversion is mediated by bacteriophages due to addition of glucosyl and/or O-acetyl groups and by plasmid due to addition of phosphoethanolamine groups to one or more sugars of the O-antigen [4]. The O-antigen modification promotes bacterial invasion and evasion of innate immunity; both of which are essential for *S. flexneri* virulence and this might have contributed to the emergence of serotype diversity [5]. The protective immune response to *S. flexneri* mainly targets against the O-antigen hence making O-antigen as a major candidate for vaccine development [6]. However, identification of several novel *S. flexneri* serotypes in the recent years has complicated any potential O-antigen based vaccine development for *Shigella* [7].

The current approaches to *Shigella* vaccines consider *S. flexneri* serotypes 2a and 3a as the major candidates for vaccine development based on animal studies suggesting cross-protection against majority of *S. flexneri* serotypes [7]. However, *S. flexneri* serotypes 2a and 3a have not yet been shown to provide cross-protection against *S. flexneri* serotype 1c strains [8]. *S. flexneri* serotype 1c, also known as 7a serotype, was first isolated in Bangladesh in the 1980s and was identified as a provisional serotype with unique O-antigen structure comprising of a disaccharide (two glucosyl group) α-D-Glcp-(1➔ 2)-α-D-Glcp linked to O4 of the N-acetyl-glucosamine residue [9]. In the 1990s, an unrelated clone of this serotype was found to be most prevalent *S. flexneri* serotype accounting for about 40% of *S. flexneri* isolates in northern Vietnam [10]. Since then, *S. flexneri* serotype 1c has been isolated and identified in Egypt, Pakistan, China, Canada and the UK [11–13]. Therefore, the widespread distribution of *S. flexneri* serotype 1c highlights the importance of its consideration for *Shigella* vaccine development strategies.

The glucosylation in O-antigen of *S. flexneri* is mediated by bacteriophage-encoded genes arranged in an operon known as the *gtr* cluster which comprises of three genes *gtrA, gtrB and gtr(type)* [14, 15]. The mechanism of O-antigen modification in serotype 1c is unique compared to other serotypes. The addition of the first glucosyl group is mediated by *gtrI* cluster found within a cryptic SfI prophage and the addition of second glucosyl group is mediated by separate *gtrI* cluster designated as *gtrIC* which came possibly from more distantly related bacteriophage [16]. So far, little is known about *S. flexneri* serotype 1c in terms of its genome organization, pathogenic islands and the evolution. A better understanding of the pathogenic signatures of any pathogen requires the availability of higher-resolution data such as the whole genome sequence.

A large number of the perfectly repeated insertion sequences in *Shigella* species impair genome assembly using sequences generated by short-read technologies, e.g. Illumina [17, 18]. This limitation can be overcome with the use of a long-read sequencing technology such as single-molecule real-time (SMRT) sequencing of Pacific Biosciences (PacBio). However, the sequence from SMRT technology has higher error rates compared to Illumina platforms [19]. Recently, a hybrid approach of using both long-read and short-read sequencing technologies has been adopted to yield a high-quality de novo assembly of prokaryotic and eukaryotic genomes [20, 21]. To better understand the genome architecture and dynamics of *S. flexneri* serotype 1c strains, we have sequenced and assembled *S. flexneri* serotype 1c strain Y394 using hybrid methods of long-read SMRT and short-read MiSeq Sequencing technologies. We present here the unique features of Y394 genome in comparison with ten other publicly available complete genome sequences of *S*. *flexneri* (Additional file 1: Table S1) thus providing further insights into the evolution and pathogenicity of this novel serotype.

## Results

### Genome assembly

Assembly of the long-read SMRT sequences generated a single bacterial chromosome of 4,585,914 bp with 203X coverage. In common with most other *S. flexneri* strains, Y394 consisted of a large virulence plasmid of 221,307 bp and an additional small plasmid of 10,873 bp (data not shown). The SMRT sequences were refined using the MiSeq short-read data. The hybrid genome assembly generated the final circular Y394 genome of 4,584,634 bp.

### General features

The 4,584,634 bp Y394 genome comprised of 4699 coding sequences with guanine-cytosine (GC) content of 50.9% (2,334,162 of 4,584,634 bp). The number of tRNA sequences were 108, and the number of rRNA sequences were 22. The comparison of genome features show that Y394 has similar nucleotide composition and size (Table 1).

**Table 1:** General features of the *Shigella flexneri* genomes

Serotype	Strain	Length (bp)	GC (%)	CDS	tRNA	IS elements	Accessory genes^a^	Unique genes^b^
1c	Y394	4,584,634	50.9	4699	108	345	979	152
1a	288	4,698,633	50.9	4841	104	363	1121	103
2a	2457 T	4,599,354	50.9	4709	101	352	989	79
2a	301	4,607,202	50.9	4715	96	358	995	68
2a	981	4,661,157	50.9	4788	106	365	1068	27
2a	NCTC1	4,526,576	50.9	4621	97	328	901	28
4c	1205	4,683,636	50.8	4823	106	367	1103	49
5b	8401	4,574,284	50.9	4668	98	312	948	246
Xv	2002017	4,650,856	50.9	4787	103	349	1067	62
Y	2003036	4,595,814	50.9	4714	98	346	994	60
Yv	Shi06HN006	4,620,903	50.9	4761	98	372	1041	56

^a^‘The accessory genes’ refer to the total number of CDS minus the number of core genes

^b^‘The unique genes’ refer to the genes present only in a given strain

### Genome rearrangements

**Fig. 1:**
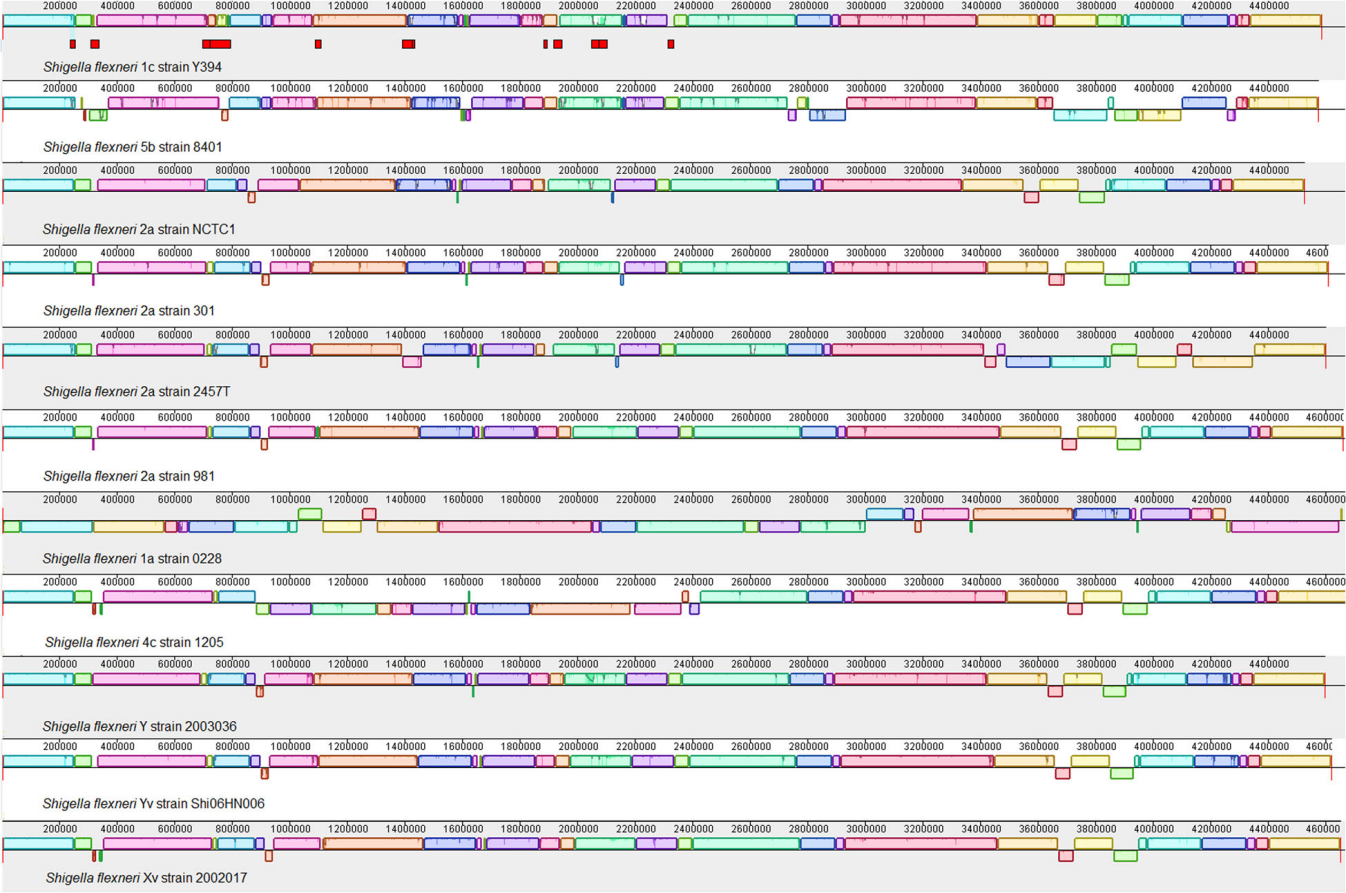
Genome-wide alignment of *Shigella flexneri* genomes. The horizontal panel of blocks from top to bottom represents the genome sequences of *S. flexneri* 1c strain Y394, *S. flexneri* 5b strain 8401, *S. flexneri* 2a strain NCTC1, *S. flexneri* 2a strain 301, *S. flexneri* 2a strain 2457 T, *S. flexneri* 2a strain 981, *S. flexneri* 1a strain 0228, *S. flexneri* 4c strain 1205, *S. flexneri* Y strain 2003036, *S. flexneri* Yv strain Shi06HN006 and *S. flexneri* Xv strain 2002017, respectively. Each syntenical placement of the homologous regions of the genomes is represented as unique coloured blocks. Blocks above and below the centre line depict the orientation of the genomic region compared to *S. flexneri* 1c strain Y394. The 12 intact prophage regions are represented by red blocks in Y394 panel. The genomes are added sequentially for comparison based on the phylogenetic distances

To examine the genome rearrangement among *S. flexneri* genomes, we performed the genome-wide alignment of 10 complete *S. flexneri* genomes with Y394 as a reference. We identified a high number of homologous genomic regions in all the compared genomes indicating high level of sequence similarity among these strains (Fig. 1). The alignment showed varying degree of genome shuffling and recombination events among different serotypes and strains of *S. flexneri*.

The GC content and GC skew varied within the Y394 genome (Additional file 2: Figure S1), which could be due to genome rearrangements and horizontal gene acquisitions. The nucleotide composition is relatively uniform over the entire bacterial genome and is conserved within related bacterial species. However, regions with anomalous GC content within a bacterial genome is likely due to recently acquired genes from distantly related organisms [22] and is a common feature of *S. flexneri* strains [20].

### Mobile genetic elements

Similar to other *S. flexneri* completed genomes (Table 1), the total number of predicted insertion sequence (IS) elements in Y394 genome was 345. The ORFs related to IS elements covered approximately 7% (311,229 bp of 4,584,634 bp) of the total genome with IS3 (47%) and IS1 (33%) being the most common IS elements (Additional file 3: Table S2).

**Fig. 2:**
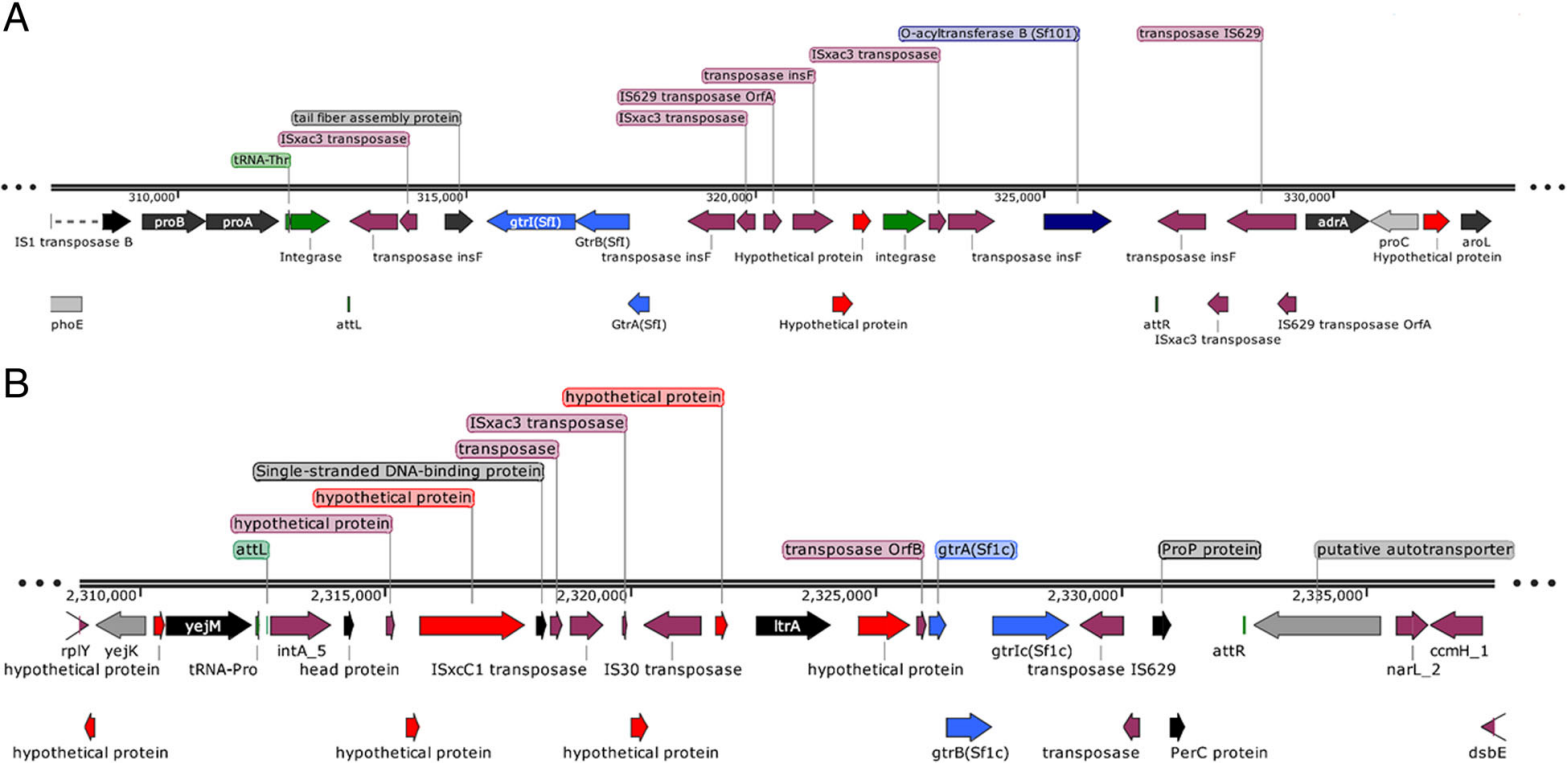
Linear schematic representation of *gtr* gene clusters in Y394. The direction of arrows indicates the orientation of open reading frames (ORFs). The color code denotes the ORF types: red, hypothetical protein; plum, transposase and IS elements, light green, tRNA; dark green, integrase; gray/black, annotated protein and blue, *gtr* genes. **a** *gtrI* encoding bacteriophage region with *oacB* gene in Y394. **b** *gtrIC* encoding bacteriophage region in Y394

We identified 12 different regions of intact prophage distributed across the genome comprising of 400 CDS with an average GC content of 49.62% (Range: 47.73–52.60) accounting up to 8% (365 kb) of the genome. There were several additional regions with incomplete (5 regions, 45.7 kb) and putative prophage regions (5 regions, 86.9 kb) (Additional file 4: Table S3). However, all of these prophages have undergone massive loss of functional genes resulting in bacteriophage genome fragmentation and ultimately resulting into cryptic prophage. Interestingly, both *gtrI* and *gtrIC* gene clusters were found to be present in two different prophage regions which were 2 Mb apart. Both these clusters were lacking phage structural components required for their replication and lysis. Another interesting finding was the identification of functional O-acyltransferase B (*oacB*) gene 6.8 kb upstream to *gtrA* gene within the *gtrI* gene cluster. The functionality of *oacB* gene was confirmed by slide agglutination assay using specific antiserum against a 3/4-O-acetylated Rha^III^ epitope prepared as described previously [23]. The analysis of *gtrI* region revealed that the cryptic phage integrated within the *proA-adrA* locus of the bacterial chromosome similar to several other *S. flexneri* serotype-converting phages [24]. The amino acid comparison of *oacB* gene from Y394 showed 100% sequence coverage and 99% identity (with 3 amino acid difference) to that of Sf101 phage which was identified in different strains of the serotype 1c [25]. We also found that all the three genes, *gtrA, gtrB* and *gtrI* of the *gtrI* operon, were highly conserved with only one point mutation (*gtrI* gene Ieu 196 Phe) as that of prophage SfI [26]. Both *gtrI* and *gtrIC* regions comprised of several transposases and phage hypothetical proteins (Fig. 2a and b).

### Pathogenicity islands in Y394 genome

Pathogenicity islands (PAIs) are the clusters of mobile genetic elements that encode virulence factors and are present in pathogenic bacterial strains but not present in related non-pathogenic strains [27]. So far, three distinct PAIs have been identified in *S. flexneri* [28]. The first PAI is the bacteriophage-encoded genes involved in serotype conversion, referred as SHI-O. The SHI-O Island in Y394 comprises 14 kb of *gtrI* gene cluster and 20 kb of *gtrIC* gene cluster.

The second PAI or SHI-1, characterized in *S. flexneri* 2a strains, encodes *sigA*, *pic* (formerly known as *she*) *set1A* and *set1B* genes [29]. These genes have been shown to be responsible for fluid accumulation in ligated rabbit ileal loops suggesting their role for the watery diarrhoea in *Shigella* infection [30, 31]. The SHI-1 is absent in Y394 genome as in several other *S. flexneri* strains of 1a, 2a, 1b, 3b, 4 and 5b serotypes [20, 32, 33]. (Additional file 5: Table S4).

The third PAI of *S. flexneri,* SHI-2, is a 23.8 kb region downstream of *selC* locus and contains genes encoding the synthesis and transport of aerobactin, iron acquisition siderophore system, associated with increased virulence of enteric bacteria [34]. The aerobactin operon consists of first four proteins IucA-D for the siderophore which complexes with iron in the host environment. The protein IutA comprises the bacterial receptor for the iron-siderophore complex [35]. The Y394 genome showed similar composition and organization of genes in the SHI-2 PAI as in other pathogenic 2a strains of *S. flexneri* (Additional file 6: Figure S2).

The Y394 genome also possessed a 24.8 kb putative pathogenic island, which constitutes *ipaH* genes that encode effector proteins secreted via the type III secretion system as well as several transposases and hypothetical genes, which might have a role in the virulence [36].

The Y394 was found to be resistant to a number of antimicrobial agents including erythromycin, penicillin, trimethoprim/sulphamethoxazole and tetracycline. The observed phenotype was consistent with antibiotic resistant genes present in the Y394 chromosome and the small plasmid. The chromosomal *acrA*, *acrB and tolC* genes codes the tripartite antibiotic efflux pump conferring resistance of Y394 to β-lactams [37, 38] that could also result in resistance to penicillin. The erythromycin resistance in Y394 is mediated by chromosomally encoded multidrug resistant efflux pump (MdtE, MdtF and TolC) [20]. The sulphonamide and tetracycline resistance in Y394 are mediated by plasmid-borne *sul2* gene and *tetA* gene, respectively [39, 40].

### Pangenome and phylogenetics

**Fig. 3:**
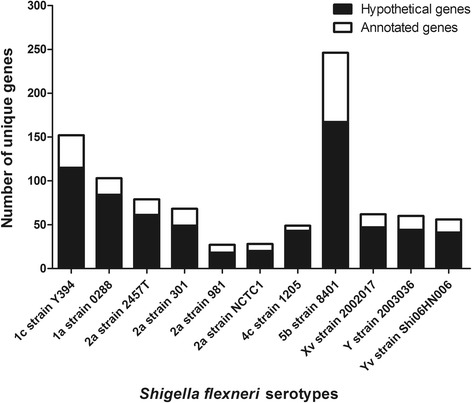
Distribution of unique genes found in the *S. flexneri* genomes. The X-axis is the *S. flexneri* serotypes, and Y-axis denotes the corresponding number of unique genes. The number of unique hypothetical genes within a genome is shaded black

The pangenome of *S. flexneri* was predicted based on the Y394 and other ten publicly available complete genome sequences of *S. flexneri.* We identified 3720 chromosomal core genes present in each of the sequenced genomes. The total pangenome of *S. flexneri* consists of 6237 genes with 2517 accessory genes. The “open” pangenome accumulation curve (Additional file 7: Figure S3) indicated that the pangenome size continues to grow as additional *S. flexneri* genomes are sequenced. The number of unique genes varied among the compared strains with an average of 84 genes (Range 27–246). Y394 genome had the highest number of unique genes (152 genes) after *S. flexneri* 5b 8401 genome (246 genes) compared with other *S. flexneri* genomes (Fig. 3).

**Fig. 4:**
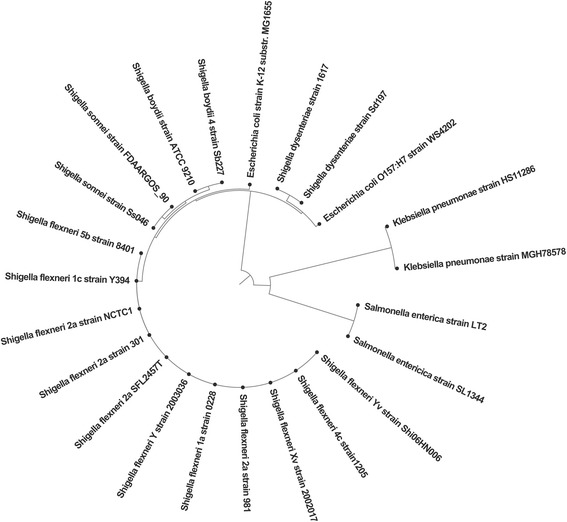
Evolutionary relationship of *Shigella flexneri.* The maximum likelihood tree of eleven *S. flexneri* genomes and other *Shigella* species, rooted with non-*Shigella* outgroups. Bootstrap support values of 1000 pseudo-replicates were 100% for all the major clades

The phylogenetic analysis was performed using 329 chromosomal genes found in all the 23 compared complete genomes including the broader *Shigella* species and representative strains of *Escherichia coli, Klebsiella pneumoniae and Salmonella enterica* (Additional file 8: Table S5). A total of 61,547 Single-nucleotide polymorphisms (SNPs) were identified within these core genes. The maximum likelihood tree generated using general time reversible (GTR) model with ascertainment bias correction (ASC) and gamma correction (with *n* = 4 categories) for site rate variation, identified three major phylogroups (Fig. 4). All the *S. flexneri* strains grouped together into a single cluster forming a phylogroup with other *Shigella* species and *E. coli*. The Y394 genome was evolutionarily more closely related to *S. flexneri* serotype 5b strain 8401 and *S. flexneri* serotype 2a strain NCTC1 (Additional file 9: Figure S4). The distantly related species *K. pneumoniae* and *S. enterica* formed an outgroup from the *Shigella* phylogroup as expected.

## Discussion

This study represents the first complete genome analysis of *S. flexneri* serotype 1c which is an emerging *S. flexneri* serotype in developing countries. Analysis of the Y394 genome revealed that mobile genetic elements including bacteriophages, IS elements, PAIs and plasmids are the driving force for bacterial diversification, adaptation and evolution, and they play an important role in pathogen virulence and spread of drug resistance. The analysis also identified unique and highly modified O-antigen structure comprising of three distinct O-antigen modifying gene clusters (*gtrI*, *gtrIC* and *oacB*) which potentially came from three different bacteriophages. The *gtrI* gene cluster located at the *proA* locus mediates the addition of the first glucosyl group and the *gtrIC* gene cluster mediates the addition of the second glucosyl group resulting into the serotype 1c specific O-antigen modification [16]. The *oacB* gene found within the *gtrI* gene cluster is responsible for the 3/4-O-acetylation in the O-antigen [25]. This highly diverse O-antigen modification in serotype 1c perhaps enhances the bacterial ability to escape the host defence mechanism. Further, the phage hypothetical proteins conserved within these clusters might have other roles in virulence. Thus, three different serotype-converting phages were acquired by Y394 and over the period have undergone massive DNA deletions resulting into cryptic phages. Hence, phages are one of the major drivers of adaptively significant genetic variation of *S. flexneri* 1c and might have roles in pathogen virulence as found in several other bacterial pathogens [41, 42].

In addition to the virulence plasmid, *S. flexneri* requires chromosomal genes for the full array of virulence phenotypes [43, 44]. Although the SHI-1 PAI was absent, there were several hypothetical genes and transposases present in the upstream region of *phe* tRNA gene, the integration site of SHI-1 PAI. The ability of the SHI-1 PAI to undergo spontaneous and precise excision via site-specific recombination [45] suggests that Y394 genome might have lost its SHI-1 region during its evolution to incorporate more important genes. The identification of the functions of these acquired genes will be interesting for further studies.

The accumulation of antimicrobial resistance determinants via horizontally acquired plasmids demonstrates how well 1c serotypes are adapted to the evolutionary pressures. The multidrug resistance in *S. flexneri* serotype 1c clinical isolates is not uncommon [11]. However, the resistance to previously efficacious first-line drugs including sulphonamides, tetracycline and trimethoprim/sulfamethoxazole used in paediatric cases is of concern [46]. The spread of multidrug resistant *S. flexneri* strain is of greater concern in developing countries due to limited laboratory settings for antibiotic susceptibility testing and unrestricted antibiotic use without proper prescription, resulting into frequent treatment failures and economic burden to underprivileged patients.

Our analysis revealed that *S. flexneri* is composed of phylogenetically distinct lineages and their genomes are relatively stable compared to other members of enterobacteriaceae family, in line with previous findings by Conner et al. [47]. The different serotypes of *S. flexneri* are classified based on the O-antigen modification resulted by the genes acquired by horizontally transmissible genetic elements such as bacteriophage and the plasmids. Therefore, it is apparent that the phylogeny based on the core genes cannot segregate the *S. flexneri* serotypes.

Acquisition of new traits by horizontal gene transfer enables bacterial pathogens to adapt to evolutionary pressures. The cryptic prophages constitute a significant amount of Y394 genome with several hypothetical genes in them. Many prophages carry additional cargo genes in their genomes which are not necessary for the phage but can change the virulence phenotype or fitness of the lysogen leading to the emergence of new pathogens or epidemic clones [41]. As the advancement in sequencing and molecular methods, the list of phage-encoded virulence genes is rapidly growing. Several bacteriophages encoded virulence factors have been identified that play a role in different stages of the bacterial pathogenesis including toxin production, host epithelial cell invasion, adhesion, intracellular survival and altering of antigenicity [42, 48, 49]. The clear understanding of the role of these unknown bacteriophage genes in the survival of *S. flexneri* serotype 1c in the human host can pave the way to the identification of potential attenuation targets and vaccine candidate antigens.

## Conclusions

Although a dozen of *S. flexneri* strains have already been sequenced, for the first time, we have sequenced the complete genome of *S. flexneri* serotype 1c (strain Y394) using hybrid sequencing approach and produced a high-quality reference genome. *S. flexneri* serotype 1c is an important serotype responsible for more recent shigellosis outbreaks in the developing nations. Our findings showed that the genome of Y394 is highly complex with a large number of unique genes acquired via horizontal gene transfer, which might be important for the pathogenesis and virulence of the pathogen. The overall genomic organization, gene contents and order are similar in all the *S. flexneri* genomes. However, the collinearity is disrupted by transposons mediated inversions. There are variations in the PAIs, more notably being SHI-O (containing O-antigen) in different *S. flexneri* genomes. The genes present in both the chromosome and the plasmid confer the resistance of the Y394 to antimicrobial agents. The identification of several hypothetical genes in the PAIs and putative bacteriophage regions warrants future investigation for determining the role of these unknown genes in relevance to pathogen’s virulence and survival in the host environment.

## Methods

### Bacterial strain and laboratory methods

*S. flexneri* serotype 1c strain Y394 was a clinical isolate from Bangladesh and kindly gifted by Nils I. A. Carlin [9]. The Y394 was grown aerobically (180 rpm) at 37 °C in Luria Bertani broth (LB). Bacterial DNA was extracted using the Genomic Tip 100/G (Qiagen) according to the manufacturer’s instructions.

The antibiotic susceptibility pattern of Y394 was determined using disk diffusion method (Kirby-Bauer) [50]. The antibiotic discs (Oxoid, UK) used in this study were ampicillin (10 μg), cefoxitin (30 μg), chloramphenicol (30 μg), erythromycin (30 μg), kanamycin (30 μg), nitrofurantoin (300 μg), penicillin (1 U), tetracycline (30 μg) and trimethoprim/sulfamethoxazole (1.25/23.75 μg).

### Whole genome sequencing

Genome sequencing of Y394 was performed using both SMRT sequencing (PacBio) and MiSeq sequencing (Illumina). The SMRTbell Template Prep Kit 1.0 (PacBio) was used for SMRT sequencing library preparation of about 20 kb insert size. The sequencing was performed using PacBio RSII sequencing system. The Nextera XT DNA library preparation kit (Illumina) was used for MiSeq v3 300 bp paired-end sequencing. Both the sequencing and library preparation was performed at the Ramaciotti Centre for Genomics, The University of New South Wales, Australia.

### Genome data and assembly

The PacBio sequencing was performed using 240 min movies (Using P6-C4 chemistry) that generated 77,493 individual reads (sub reads) of DNA fragment with N50 of 20.6 kb. A de novo assembly of these reads was performed with HGAP.3 (Pacific Biosciences) on the SMRT Analysis Pipeline version 2.3.0 [51]. The Y394 genome was obtained as a contiguous sequence of 4,585,914 bp with 203X coverage.

The MiSeq sequencing generated 2 × 338,140 paired-end reads of Y394 genome with the average fragment size of 291 bp (range 55–301). The quality of the reads was assessed using FastQC v0.11.5 [52]. The initial 20 bp and beyond 200 bp of each read were trimmed to obtain high quality bases using Trimmomatic v0.36 [53]. The assembly of the trimmed reads was performed using the VelvetOptimiser Script [54].

BWA-MEM [55] was used to map the trimmed MiSeq reads against the PacBio based genome assembly followed by the use of Pilon for PacBio sequence assembly improvement [56, 57]. The sequence assembly pipeline adapted from [58] is outlined in Additional file 10: Figure S5.

### Sequence analysis

The annotations of the final Y394 genome as well as other completed genomes of *S. flexneri* included in this study were performed using Rapid Annotation of Prokaryotic Genomes (prokka) [59]. The prokka annotation was re-annotated with PHASTER (PHAge Search Tool - Enhanced Release) database [60] to find the bacteriophage-encoded regions in the Y394 chromosome. The genome-scale alignments were performed using Mauve alignment Tool [61] and CG View Server [62]. The IS elements were identified using ISsaga [63]. The antibiotic resistance profile was generated using ARDB-Antibiotic Resistance Genes Database [64].

The pangenome of *S. flexneri* was predicted based on the completed genomes of different strains of *S. flexneri*. All these genomes were re-annotated using prokka to avoid biases in the comparisons due to different annotations. The pangenome analysis was performed using Roary Pipeline [65]. The phylogenetic tree was constructed based on the 329 core genes present in all the compared *S. flexneri* genomes and non-*S. flexneri* genomes including representative strains of other *Shigella* species, *E. coli, K. pneumoniae* and *S. enterica*. The 61,547 SNP sites were identified and concatenated to form a multiple alignment of SNPs for phylogenetic analysis using the package SNP-sites [66]. The best-fit substitution model was determined (GTR + ASC + G4) and used for constructing the maximum likelihood tree using the package IQ-tree [67]. The bootstrap analysis was performed using 1000 randomizations. The image files were generated using Snap Gene Viewer (Version 3.3.1), GraphPad Prism (Version 5.01) and Fig Tree (version 1.4.3) [68].

## Additional files

Additional file 1: Table S1. List of complete *S. flexneri* genomes and their accession numbers used for comparative genomics. (PDF 7 kb)

Additional file 2: Figure S1. Schematic Circular genome map of the Y394. The two outer rings represent ORFs encoded by leading and lagging strands with color codes depicting the genome features- blue, CDS; dark red, tRNA and pink, rRNA. The third ring with green bands represent the 12 intact prophage regions. The fourth ring in black shows the deviation from the average percentage (G + C) content. The green and purple color of the fifth ring represents the GC skew (G-C/G + C). The innermost ring represents the nucleotide position in the genome. (PDF 653 kb)

Additional file 3: Table S2. Predicted Insertion Sequence (IS) family in Y394 genome. (PDF 12 kb)

Additional file 4: Table S3. Prophage regions identified in Y394 genome. (PDF 27 kb)

Additional file 5: Table S4. Pathogenicity Islands in *Shigella flexneri* genomes. (PDF 158 kb)

Additional file 6: Figure S2. Linear schematic representation of SHI-2 pathogenicity Island in Y394. (PDF 69 kb)

Additional file 7: Figure S3. Pangenome accumulation curve. The X- axis indicates the total number of genomes and Y-axis shows the number of genes- conserved vs total gene in *S. flexneri* pangenome. (PDF 34 kb)

Additional file 8: Table S5. List of complete genomes and their accession numbers of bacterial strains used for phylogenetics. (PDF 12 kb)

Additional file 9: Figure S4. The phylogenetic relationship of *Shigella flexneri.* The maximum likelihood tree of eleven *Shigella flexneri* genomes based on 6387 SNP sites of the 3720 core genes. The numbers represent the bootstrap support values of 1000 pseudo-replicates. (PDF 32 kb)

Additional file 10: Figure S5. Flowchart depicting PacBio-Miseq hybrid genome assembly. The arrows indicate the sequential steps used for assembly. (PDF 25 kb)

## Abbreviations

GC: Guanine and cytosine
IS: Insertion sequences
PAI: Pathogenicity islands
SMRT: Single molecule real-time
SNP: Single-nucleotide polymorphism

## References

[1] Bardhan P, Faruque AS, Naheed A, Sack DA (2010). Decrease in shigellosis-related deaths without Shigella spp.-specific interventions, Asia. Emerg Infect Dis.

[2] Gu B, Cao Y, Pan S, Zhuang L, Yu R, Peng Z, Qian H, Wei Y, Zhao L, Liu G (2012). Comparison of the prevalence and changing resistance to nalidixic acid and ciprofloxacin of Shigella between Europe-America and Asia-Africa from 1998 to 2009. Int J Antimicrob Agents.

[3] Sun Q, Lan R, Wang J, Xia S, Wang Y, Wang Y, Jin D, Yu B, Knirel YA, Xu J (2013). Identification and characterization of a novel Shigella flexneri serotype Yv in China. PLoS One.

[4] Allison GE, Verma NK (2000). Serotype-converting bacteriophages and O-antigen modification in Shigella flexneri. Trends Microbiol.

[5] West NP, Sansonetti P, Mounier J, Exley RM, Parsot C, Guadagnini S, Prevost MC, Prochnicka-Chalufour A, Delepierre M, Tanguy M (2005). Optimization of virulence functions through glucosylation of Shigella LPS. Science.

[6] Thanh DP, Holt KE, Thomson NR, Baker S, The HC (2016). The genomic signatures of Shigella evolution, adaptation and geographical spread. Nat Rev Microbiol.

[7] Walker RI (2015). An assessment of enterotoxigenic Escherichia Coli and Shigella vaccine candidates for infants and children. Vaccine.

[8] Noriega FR, Liao FM, Maneval DR, Ren S, Formal SB, Levine MM (1999). Strategy for cross-protection among Shigella flexneri serotypes. Infect Immun.

[9] Wehler T, Carlin NI (1988). Structural and immunochemical studies of the lipopolysaccharide from a new provisional serotype of Shigella flexneri. Eur J Biochem.

[10] Stagg RM, Cam PD, Verma NK (2008). Identification of newly recognized serotype 1c as the most prevalent Shigella flexneri serotype in northern rural Vietnam. Epidemiol Infect.

[11] Ahmed SF, Klena J, Husain T, Monestersky J, Naguib A, Wasfy MO (2013). Genetic characterization of antimicrobial resistance of Shigella flexneri 1c isolates from patients in Egypt and Pakistan. Ann Clin Microbiol Antimicrob.

[12] El-Gendy A, El-Ghorab N, Lane EM, Elyazeed RA, Carlin NI, Mitry MM, Kay BA, Savarino SJ, Peruski LF (1999). Identification of Shigella flexneri subserotype 1c in rural Egypt. J Clin Microbiol.

[13] Qiu S, Xu X, Wang Y, Yang G, Wang Z, Wang H, Zhang L, Liu N, Chen C, Liu W (2012). Emergence of resistance to fluoroquinolones and third-generation cephalosporins in Shigella flexneri subserotype 1c isolates from China. Clin Microbiol Infect.

[14] Mavris M, Manning PA, Morona R (1997). Mechanism of bacteriophage SfII-mediated serotype conversion in Shigella flexneri. Mol Microbiol.

[15] Guan S, Bastin DA, Verma NK (1999). Functional analysis of the O antigen glucosylation gene cluster of Shigella flexneri bacteriophage SfX. Microbiology.

[16] Stagg RM, Tang SS, Carlin NI, Talukder KA, Cam PD, Verma NK (2009). A novel glucosyltransferase involved in O-antigen modification of Shigella flexneri serotype 1c. J Bacteriol.

[17] Scheibye-Alsing K, Hoffmann S, Frankel A, Jensen P, Stadler PF, Mang Y, Tommerup N, Gilchrist MJ, Nygard AB, Cirera S (2009). Sequence assembly. Comput Biol Chem.

[18] Jin Q, Yuan Z, Xu J, Wang Y, Shen Y, Lu W, Wang J, Liu H, Yang J, Yang F (2002). Genome sequence of Shigella flexneri 2a: insights into pathogenicity through comparison with genomes of Escherichia Coli K12 and O157. Nucleic Acids Res.

[19] Quail MA, Smith M, Coupland P, Otto TD, Harris SR, Connor TR, Bertoni A, Swerdlow HP, Gu Y (2012). A tale of three next generation sequencing platforms: comparison of ion torrent, Pacific Biosciences and Illumina MiSeq sequencers. BMC Genomics.

[20] Baker KS, Mather AE, McGregor H, Coupland P, Langridge GC, Day M, Deheer-Graham A, Parkhill J, Russell JE, Thomson NR (2014). The extant world war 1 dysentery bacillus NCTC1: a genomic analysis. Lancet.

[21] Koren S, Schatz MC, Walenz BP, Martin J, Howard JT, Ganapathy G, Wang Z, Rasko DA, McCombie WR, Jarvis ED (2012). Hybrid error correction and de novo assembly of single-molecule sequencing reads. Nat Biotechnol.

[22] Lawrence JG, Ochman H (1997). Amelioration of bacterial genomes: rates of change and exchange. J Mol Evol.

[23] Freter R (1957). Agglutinating efficiency and combining capacity of Shigella and vibrio antisera from rabbits at different stages of immunization. J Exp Med.

[24] Wang J, Knirel YA, Lan R, Senchenkova SN, Luo X, Perepelov AV, Wang Y, Shashkov AS, Xu J, Sun Q (2014). Identification of an O-acyltransferase gene (oacB) that mediates 3- and 4-O-acetylation of rhamnose III in Shigella flexneri O antigens. J Bacteriol.

[25] Jakhetia R, Marri A, Stahle J, Widmalm G, Verma NK (2014). Serotype-conversion in Shigella flexneri: identification of a novel bacteriophage, Sf101, from a serotype 7a strain. BMC Genomics.

[26] Sun Q, Lan R, Wang J, Wang Y, Li P, Du P, Xu J. Isolation and genomic characterization of SfI, a serotype-converting bacteriophage of Shigella flexneri. BMC Microbiol. 2013;13:39. 10.1186/1471-2180-13-39 PMC3636060 23414301

[27] Hacker J, Blum-Oehler G, Muhldorfer I, Tschape H (1997). Pathogenicity islands of virulent bacteria: structure, function and impact on microbial evolution. Mol Microbiol.

[28] Walker JC, Verma NK (2002). Identification of a putative pathogenicity island in Shigella flexneri using subtractive hybridisation of the S. Flexneri and Escherichia Coli genomes. FEMS Microbiol Lett.

[29] Rajakumar K, Sasakawa C, Adler B (1997). Use of a novel approach, termed island probing, identifies the Shigella flexneri she pathogenicity island which encodes a homolog of the immunoglobulin a protease-like family of proteins. Infect Immun.

[30] Fasano A, Noriega FR, Liao FM, Wang W, Levine MM (1997). Effect of shigella enterotoxin 1 (ShET1) on rabbit intestine in vitro and in vivo. Gut.

[31] Henderson IR, Czeczulin J, Eslava C, Noriega F, Nataro JP (1999). Characterization of pic, a secreted protease of Shigella flexneri and enteroaggregative Escherichia Coli. Infect Immun.

[32] Al-Hasani K, Adler B, Rajakumar K, Sakellaris H (2001). Distribution and structural variation of the she pathogenicity island in enteric bacterial pathogens. J Med Microbiol.

[33] Nie H, Yang F, Zhang X, Yang J, Chen L, Wang J, Xiong Z, Peng J, Sun L, Dong J (2006). Complete genome sequence of Shigella flexneri 5b and comparison with Shigella flexneri 2a. BMC Genomics.

[34] Vokes SA, Reeves SA, Torres AG, Payne SM (1999). The aerobactin iron transport system genes in Shigella flexneri are present within a pathogenicity island. Mol Microbiol.

[35] Moss JE, Cardozo TJ, Zychlinsky A, Groisman EA (1999). The selC-associated SHI-2 pathogenicity island of Shigella flexneri. Mol Microbiol.

[36] Ashida H, Toyotome T, Nagai T, Sasakawa C (2007). Shigella chromosomal IpaH proteins are secreted via the type III secretion system and act as effectors. Mol Microbiol.

[37] Daury L, Orange F, Taveau JC, Verchere A, Monlezun L, Gounou C, Marreddy RK, Picard M, Broutin I, Pos KM (2016). Tripartite assembly of RND multidrug efflux pumps. Nat Commun.

[38] Ma D, Cook DN, Alberti M, Pon NG, Nikaido H, Hearst JE (1995). Genes acrA and acrB encode a stress-induced efflux system of Escherichia Coli. Mol Microbiol.

[39] Iqbal MS, Rahman M, Islam R, Banik A, Amin MB, Akter F, Talukder KA (2014). Plasmid-mediated sulfamethoxazole resistance encoded by the sul2 gene in the multidrug-resistant Shigella flexneri 2a isolated from patients with acute diarrhea in Dhaka, Bangladesh. PLoS One.

[40] Toro CS, Farfan M, Contreras I, Flores O, Navarro N, Mora GC, Prado V (2005). Genetic analysis of antibiotic-resistance determinants in multidrug-resistant Shigella strains isolated from Chilean children. Epidemiol Infect.

[41] Brussow H, Canchaya C, Hardt WD (2004). Phages and the evolution of bacterial pathogens: from genomic rearrangements to lysogenic conversion. Microbiol Mol Biol Rev.

[42] Boyd EF (2012). Bacteriophage-encoded bacterial virulence factors and phage-pathogenicity island interactions. Adv Virus Res.

[43] Ingersoll M, Groisman EA, Zychlinsky A (2002). Pathogenicity islands of Shigella. Curr Top Microbiol Immunol.

[44] Schmidt H, Hensel M (2004). Pathogenicity islands in bacterial pathogenesis. Clin Microbiol Rev.

[45] Sakellaris H, Luck SN, Al-Hasani K, Rajakumar K, Turner SA, Adler B (2004). Regulated site-specific recombination of the she pathogenicity island of Shigella flexneri. Mol Microbiol.

[46] Ghosh S, Pazhani GP, Chowdhury G, Guin S, Dutta S, Rajendran K, Bhattacharya MK, Takeda Y, Niyogi SK, Nair GB (2011). Genetic characteristics and changing antimicrobial resistance among Shigella spp. isolated from hospitalized diarrhoeal patients in Kolkata, India. J Med Microbiol.

[47] Connor TR, Barker CR, Baker KS, Weill FX, Talukder KA, Smith AM, Baker S, Gouali M, Pham Thanh D, Jahan Azmi I (2015). Species-wide whole genome sequencing reveals historical global spread and recent local persistence in Shigella flexneri. elife.

[48] Barondess JJ, Beckwith J (1990). A bacterial virulence determinant encoded by lysogenic coliphage lambda. Nature.

[49] Waldor MK, Mekalanos JJ (1996). Lysogenic conversion by a filamentous phage encoding cholera toxin. Science.

[50] Cavalieri SJ, Harbeck RJ, YS MC, Ortez JH, Rankin ID, Sautter RL, Sharp SE, Spiegel CA. Manual of Antimicrobial Susceptibility Testing. Seattle, Washington 98195. American Society for Microbiology. 2005.

[51] Chin CS, Alexander DH, Marks P, Klammer AA, Drake J, Heiner C, Clum A, Copeland A, Huddleston J, Eichler EE (2013). Nonhybrid, finished microbial genome assemblies from long-read SMRT sequencing data. Nat Methods.

[52] FastQC High Throughput Sequence QC Report. [http://www.bioinformatics.babraham.ac.uk/projects/fastqc/].

[53] Bolger AM, Lohse M, Usadel B (2014). Trimmomatic: a flexible trimmer for Illumina sequence data. Bioinformatics.

[54] Zerbino DR. Using the Velvet de novo assembler for short-read sequencing technologies. Curr Protoc Bioinformatics. 2010; Chapter 11:Unit 11 15 10.1002/0471250953.bi1105s31 PMC2952100 20836074

[55] Li H: Aligning sequence reads, clone sequences and assembly contigs with BWA-MEM. In*.*, vol. 0: The Oxford University Press; 2013: 1–3.

[56] Li H, Durbin R (2010). Fast and accurate long-read alignment with burrows-wheeler transform. Bioinformatics.

[57] Walker BJ, Abeel T, Shea T, Priest M, Abouelliel A, Sakthikumar S, Cuomo CA, Zeng Q, Wortman J, Young SK (2014). Pilon: an integrated tool for comprehensive microbial variant detection and genome assembly improvement. PLoS One.

[58] PacBio reads: Assembly with command line tools. [https://sepsis-omics.github.io/tutorials/modules/cmdline_assembly/].

[59] Seemann T (2014). Prokka: rapid prokaryotic genome annotation. Bioinformatics.

[60] Arndt D, Grant JR, Marcu A, Sajed T, Pon A, Liang Y, Wishart DS (2016). PHASTER: a better, faster version of the PHAST phage search tool. Nucleic Acids Res.

[61] Darling AC, Mau B, Blattner FR, Perna NT (2004). Mauve: multiple alignment of conserved genomic sequence with rearrangements. Genome Res.

[62] Grant JR, Stothard P (2008). The CGView Server: a comparative genomics tool for circular genomes. Nucleic Acids Res.

[63] Varani AM, Siguier P, Gourbeyre E, Charneau V, Chandler M (2011). ISsaga is an ensemble of web-based methods for high throughput identification and semi-automatic annotation of insertion sequences in prokaryotic genomes. Genome Biol.

[64] Liu B, Pop M (2009). ARDB--antibiotic resistance genes database. Nucleic Acids Res.

[65] Page AJ, Cummins CA, Hunt M, Wong VK, Reuter S, Holden MT, Fookes M, Falush D, Keane JA, Parkhill J (2015). Roary: rapid large-scale prokaryote pan genome analysis. Bioinformatics.

[66] Page AJ, Taylor B, Delaney AJ, Soares J, Seemann T, Keane JA, Harris SR (2016). SNP-sites: rapid efficient extraction of SNPs from multi-FASTA alignments. Microbial Genomics.

[67] Nguyen L-T, Schmidt HA, von Haeseler A, Minh BQ (2015). IQ-TREE: a fast and effective stochastic algorithm for estimating maximum-likelihood phylogenies. Mol Biol Evol.

[68] FigTree [http://tree.bio.ed.ac.uk/software/figtree/].

