# Supplementary material for: Bacteriophages are the major drivers of Shigella flexneri serotype 1c genome plasticity: a complete genome analysis
Source: BMC Genomics. 2017 Sep 12;18:722. doi: 10.1186/s12864-017-4109-4 (PMC5596473; doi:10.1186/s12864-017-4109-4)
Supplement: Supplementary file 1 — List of complete S. flexneri genomes and their accession numbers used for comparative genomics. (PDF 7 kb) [file 12864_2017_4109_MOESM1_ESM.pdf]

**Table S1. List of complete *Shigella flexneri* genomes and their accession numbers used for comparative genomics.**

| S.N | *Shigella flexneri* Types (serotype and strain) | Accession Numbers |
|---|---|---|
| **1** | *Shigella flexneri* 1a strain 0228 | CP012735.1 |
| **2** | *Shigella flexneri* 2a strain 2457T | AE014073.1 |
| **3** | *Shigella flexneri* 2a strain 301 | AE005674.2 |
| **4** | *Shigella flexneri* 2a strain 981 | CP012137.1 |
| **5** | *Shigella flexneri* 2a strain NCTC1 | LM651928.1 |
| **6** | *Shigella flexneri* 4c strain 1205 | CP012140.1 |
| **7** | *Shigella flexneri* 5b strain 8401 | CP000266.1 |
| **8** | *Shigella flexneri* Xv 2002017 | CP001383.1 |
| **9** | *Shigella flexneri* Y strain 2003036 | CP004056.1 |
| **10** | *Shigella flexneri* Yv strain Shi06HN006 | CP004057.1 |
