# Supplementary material for: Bacteriophages are the major drivers of Shigella flexneri serotype 1c genome plasticity: a complete genome analysis
Source: BMC Genomics. 2017 Sep 12;18:722. doi: 10.1186/s12864-017-4109-4 (PMC5596473; doi:10.1186/s12864-017-4109-4)
Supplement: Supplementary file 2 — Schematic Circular genome map of the Y394. The two outer rings represent ORFs encoded by leading and lagging strands with color codes depicting the genome features- blue, CDS; dark red, tRNA and pink, rRNA. The third ring with green bands represent the 12 intact prophage regions. The fourth ring in black shows the deviation from the average percentage (G + C) content. The green and purple color of the fifth ring represents the GC skew (G-C/G + C). The innermost ring represents the nucleotide position in the genome. (PDF 653 kb) [file 12864_2017_4109_MOESM2_ESM.pdf]

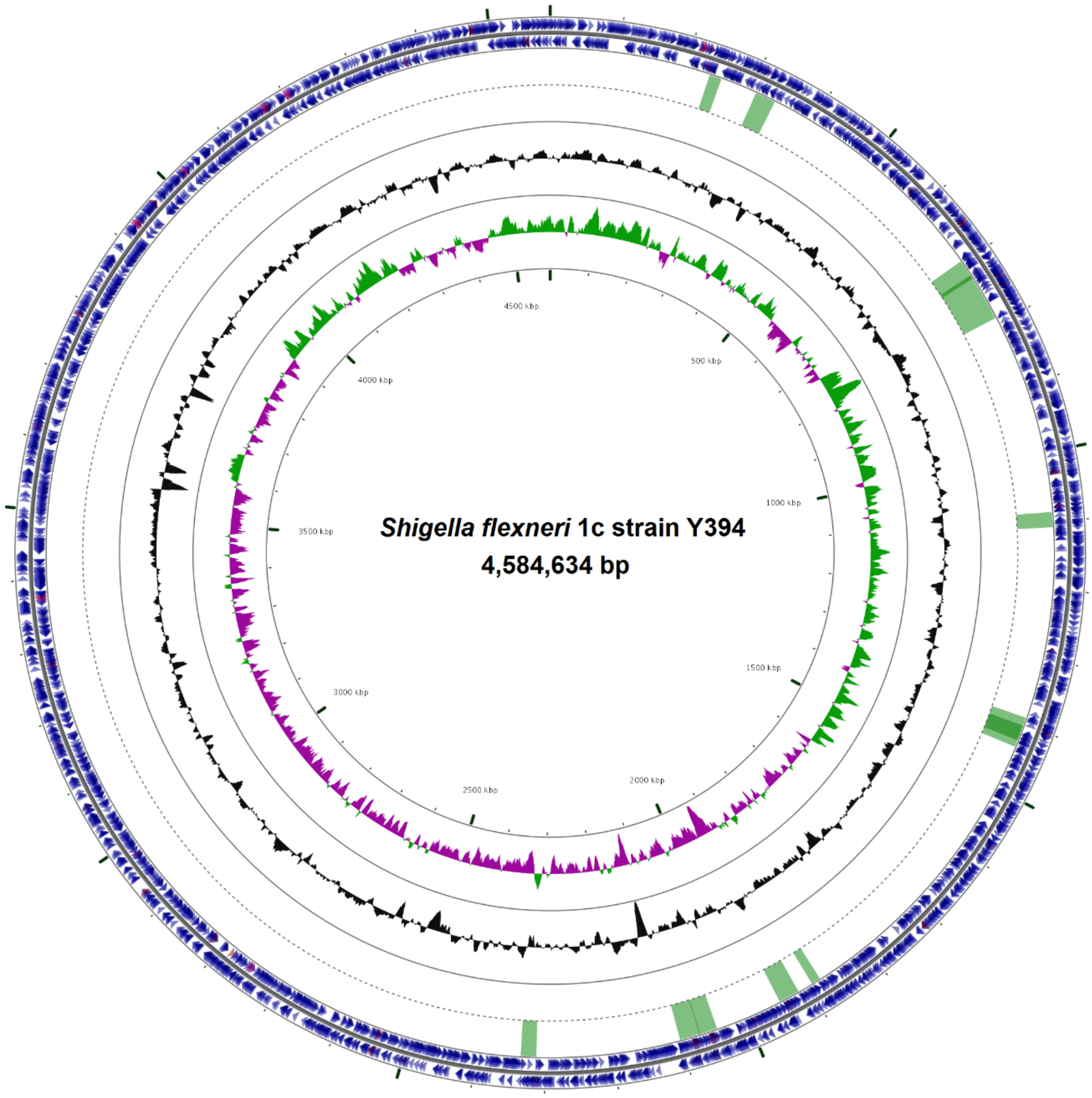

Figure S1: Schematic Circular genome map of the Y394. The two outer rings represent ORFs encoded by leading and lagging strands with color codes depicting the genome features- blue, CDS; dark red, tRNA and pink, rRNA. The third ring with green bands represent the 12 intact prophage regions. The fourth ring in black shows the deviation from the average percentage (G+C) content. The green and purple color of the fifth ring represents the GC skew (G-C/G+C). The innermost ring represents the nucleotide position in the genome.
