# Supplementary material for: Bacteriophages are the major drivers of Shigella flexneri serotype 1c genome plasticity: a complete genome analysis
Source: BMC Genomics. 2017 Sep 12;18:722. doi: 10.1186/s12864-017-4109-4 (PMC5596473; doi:10.1186/s12864-017-4109-4)
Supplement: Supplementary file 3 — Predicted Insertion Sequence (IS) family in Y394 genome. (PDF 12 kb) [file 12864_2017_4109_MOESM3_ESM.pdf]

Table S2. Predicted Insertion Sequence (IS) family in Y394 genome

IS Family	Different IS(s)	Total IS(s)
**IS1**	2	115
**IS110**	2	7
**IS200_IS605**	2	8
**IS3_ssgr_IS150**	3	7
**IS3_ssgr_IS2**	1	41
**IS3_ssgr_IS3**	5	89
**IS3_ssgr_IS407**	1	1
**IS3_ssgr_IS51**	3	25
**IS30**	1	1
**IS4_ssgr_IS4**	1	24
**IS4_ssgr_IS50**	1	1
**IS66**	1	10
**IS91**	2	10
**ISL3**	3	3
**ISNCY_ssgr_ISPlu15**	4	4
