# Supplementary material for: Bacteriophages are the major drivers of Shigella flexneri serotype 1c genome plasticity: a complete genome analysis
Source: BMC Genomics. 2017 Sep 12;18:722. doi: 10.1186/s12864-017-4109-4 (PMC5596473; doi:10.1186/s12864-017-4109-4)
Supplement: Supplementary file 4 — Prophage regions identified in Y394 genome. (PDF 27 kb) [file 12864_2017_4109_MOESM4_ESM.pdf]

Table S3. Prophage regions identified in Y394 genome (12 regions are intact, 5 regions are incomplete, and 5 regions are questionable/putative).

Region	Region Length	Completeness*	# Total Proteins	Region Position	GC content(%)
**1**	17.9Kb	intact	23	235306-253205	51.38
**2**	30.8Kb	intact (*gtrI* cluster)	25	307273-338131	47.83
**3**	17.7Kb	questionable	10	480030-497755	52.14
**4**	30.1Kb	intact	25	696673-726866	49.48
**5**	71.6Kb	intact	106	721365-793060	51.13
**6**	22.3Kb	intact	16	1087374-1109698	49.45
**7**	14.5Kb	questionable	20	1181709-1196210	50.68
**8**	10Kb	incomplete	12	1359021-1369057	52.38
**9**	34.9Kb	intact	32	1390023-1424936	49.59
**10**	32.7Kb	intact	26	1402184-1434907	49.14
**11**	13.8Kb	incomplete	20	1569542-1583420	48.71
**12**	13.6Kb	intact	21	1880663-1894277	48.55
**13**	30.9Kb	intact	39	1916716-1947708	52.6
**14**	7.5Kb	incomplete	9	2033208-2040794	50.63
**15**	26.7Kb	intact	32	2046655-2073385	48.88
**16**	30.3Kb	intact	33	2072332-2102688	49.3
**17**	15.4Kb	questionable	17	2228601-2244047	50.39
**18**	22.7Kb	intact (*gtrIC* cluster)	23	2313377-2336092	48.16
**19**	8.2Kb	incomplete	8	2383283-2391544	49.9
**20**	10.5Kb	questionable	12	2708259-2718848	49.61
**21**	6.2Kb	incomplete	9	3601945-3608185	48.09
**22**	28.8Kb	questionable	17	4360722-4389577	48.61

*Completeness of the prophage regions are based on the scoring method outlined in PHASTER database.
