# Supplementary material for: Bacteriophages are the major drivers of Shigella flexneri serotype 1c genome plasticity: a complete genome analysis
Source: BMC Genomics. 2017 Sep 12;18:722. doi: 10.1186/s12864-017-4109-4 (PMC5596473; doi:10.1186/s12864-017-4109-4)
Supplement: Supplementary file 5 — Pathogenicity Islands in Shigella flexneri genomes. (PDF 158 kb) [file 12864_2017_4109_MOESM5_ESM.pdf]

Table S4. Pathogenicity Islands in *Shigella flexneri* genomes.

Pathogenicity Island	Function	*Shigella flexneri* genomes									
		1c	5b	2a				1a	4c	Y	Yv	Xv
		Position in Y394	8401	NCTC1	301	2457T	981	228	1205	2003036	Shi06HN006	2002017
**SHI-O**	Serotype Conversion											
**a. *gtr* gene cluster-1 (*gtrI*)**	Glucosylation	312,950-326,984	Present	Present	Present	Present	Present	Present	Present	Absent	Absent	Present
**b. *oacB* gene**	O-acetylation	325,007-326,179	Present	Present	Present	Present	Present	Present	Present	Absent	Present	Present
**c. *gtr* gene cluster-2 (*gtrIC*)**	Glucosylation	2,312,600-2,332,534	Absent	Absent	Absent	Absent	Absent	Absent	Absent	Absent	Absent	Absent
**SHI-1 (*she*PAI)**	Virulence (Fluid accumulation)	Absent	Absent	Absent	Present	Present	Present	Present	Present	Present	Present	Present
**SHI-2**	Iron acquisition	3,780,936-3,804,736	Present	Present	Present	Present	Present	Present	Present	Present	Present	Present
***ipaH* Island 1**	Virulence	765,178-789,980	Present	Absent	Present	Present	Present	Present	Present	Present	Present	Present
