# Supplementary material for: Bacteriophages are the major drivers of Shigella flexneri serotype 1c genome plasticity: a complete genome analysis
Source: BMC Genomics. 2017 Sep 12;18:722. doi: 10.1186/s12864-017-4109-4 (PMC5596473; doi:10.1186/s12864-017-4109-4)
Supplement: Supplementary file 7 — Pangenome accumulation curve. The X- axis indicates the total number of genomes and Y-axis shows the number of genes- conserved vs total gene in S. flexneri pangenome. (PDF 34 kb) [file 12864_2017_4109_MOESM7_ESM.pdf]

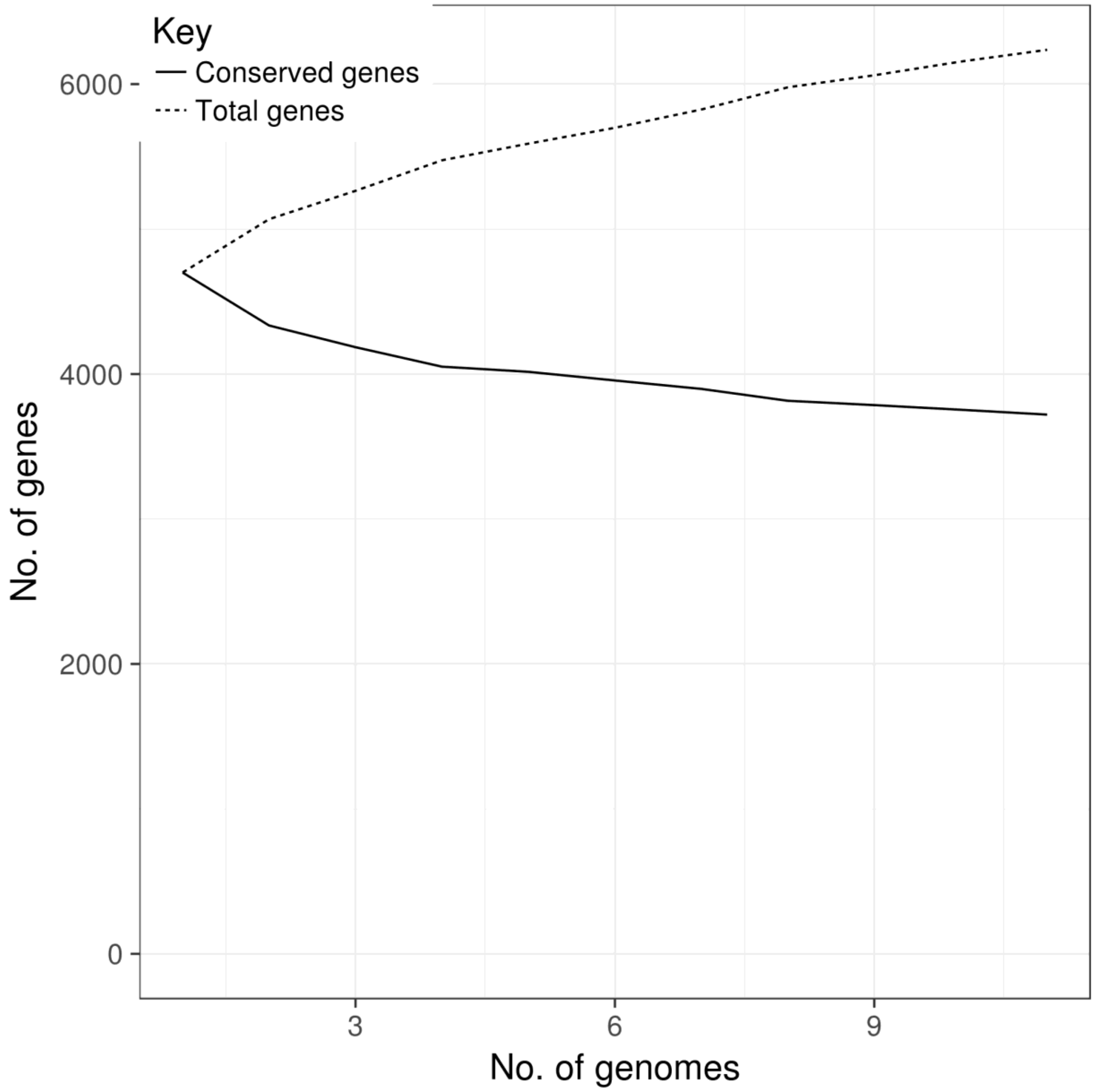

Figure S3. Pangenome accumulation curve. The X- axis indicates the total number of genomes and Y-axis shows the number of genes- conserved vs total gene in *S. flexneri* pangenome.
