# Supplementary material for: Bacteriophages are the major drivers of Shigella flexneri serotype 1c genome plasticity: a complete genome analysis
Source: BMC Genomics. 2017 Sep 12;18:722. doi: 10.1186/s12864-017-4109-4 (PMC5596473; doi:10.1186/s12864-017-4109-4)
Supplement: Supplementary file 8 — List of complete genomes and their accession numbers of bacterial strains used for phylogenetics. (PDF 12 kb) [file 12864_2017_4109_MOESM8_ESM.pdf]

Table S5. List of complete genomes and their accession numbers of bacterial strains used additionally for phylogenetics.

**S.N**	Bacterial species	**Accesson Numbers**
**1**	*Escherichia coli* O157:H7 strain WS4202	CP012802.1
**2**	*Escherichia coli* strain K-12 substr. MG1655	NC_000913.3
**3**	*Klebsiella pneumonae* strain HS11286	CP003200.1
**4**	*Klebsiella pneumonae* strain MGH 78578	NC_009648.1
**5**	Salmonella enteric*a* strain SL1344	FQ312003.1
**6**	*Salmonella enterica* strain LT2	NZ_CP014051.1
**7**	*Shigella boydii* 4 strain Sb227	CP000036.1
**8**	*Shigella boydii* strain ATCC 9210	CP011511.1
**9**	*Shigella dysenteriae* strain 1617	CP006736.1
**10**	*Shigella dysenteriae* strain Sd197	CP000034.1
**11**	*Shigella sonnei* strain FDAARGOS_90	CP014099.1
**12**	*Shigella sonnei* strain Ss046	CP000038.1
