# Supplementary material for: Bacteriophages are the major drivers of Shigella flexneri serotype 1c genome plasticity: a complete genome analysis
Source: BMC Genomics. 2017 Sep 12;18:722. doi: 10.1186/s12864-017-4109-4 (PMC5596473; doi:10.1186/s12864-017-4109-4)
Supplement: Supplementary file 9 — The phylogenetic relationship of Shigella flexneri. The maximum likelihood tree of eleven Shigella flexneri genomes based on 6387 SNP sites of the 3720 core genes. The numbers represent the bootstrap support values of 1000 pseudo-replicates. (PDF 32 kb) [file 12864_2017_4109_MOESM9_ESM.pdf]

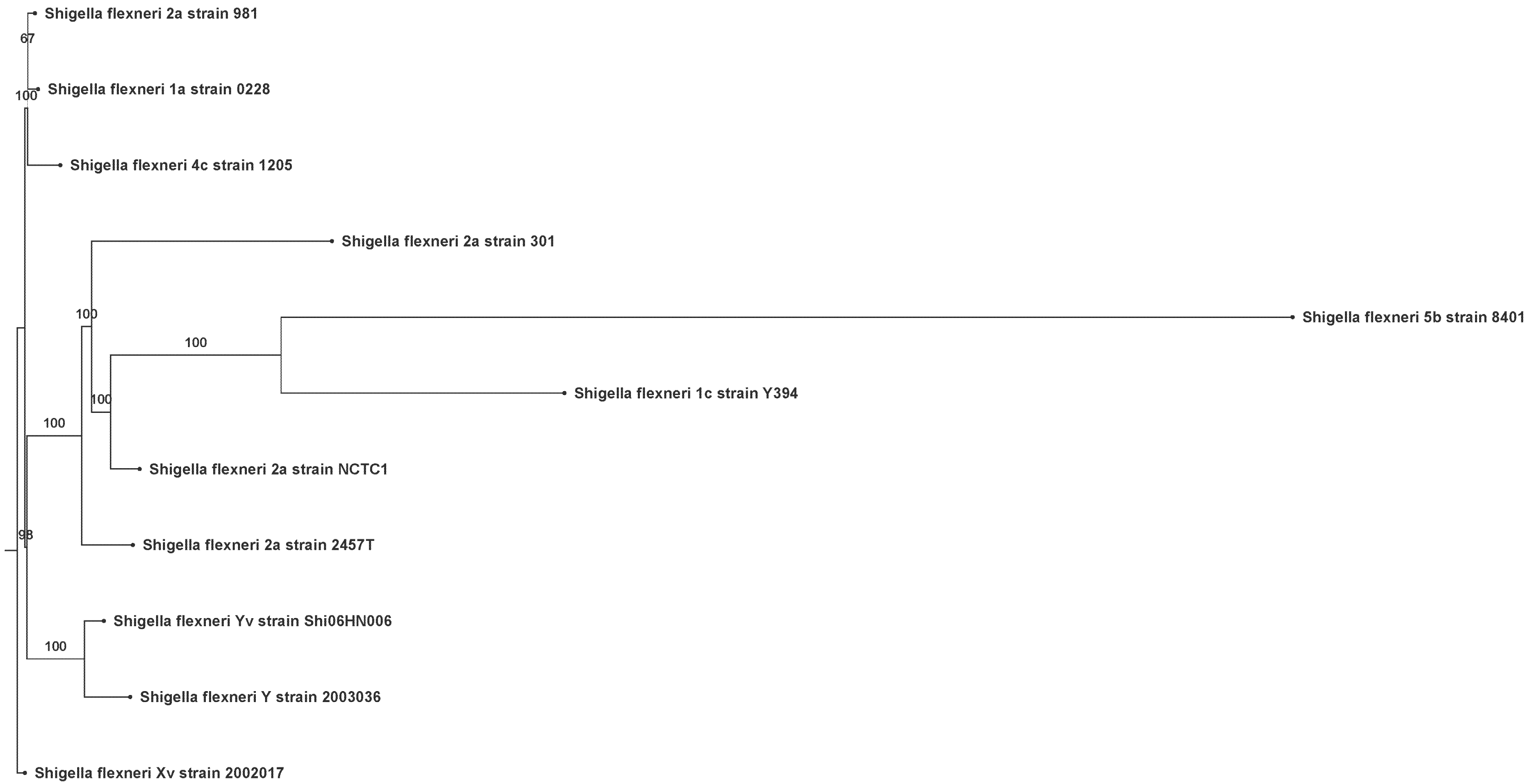

Figure S4. The phylogenetic relationship of *Shigella flexneri*. The maximum likelihood tree of eleven *Shigella flexneri* genomes based on 6,387 SNP sites of the 3,720 core genes. The numbers represent the bootstrap support values of 1000 pseudo-replicates.
