# Supplementary material for: Bacteriophages are the major drivers of Shigella flexneri serotype 1c genome plasticity: a complete genome analysis
Source: BMC Genomics. 2017 Sep 12;18:722. doi: 10.1186/s12864-017-4109-4 (PMC5596473; doi:10.1186/s12864-017-4109-4)
Supplement: Supplementary file 10 — Flowchart depicting PacBio-Miseq hybrid genome assembly. The arrows indicate the sequential steps used for assembly. (PDF 25 kb) [file 12864_2017_4109_MOESM10_ESM.pdf]

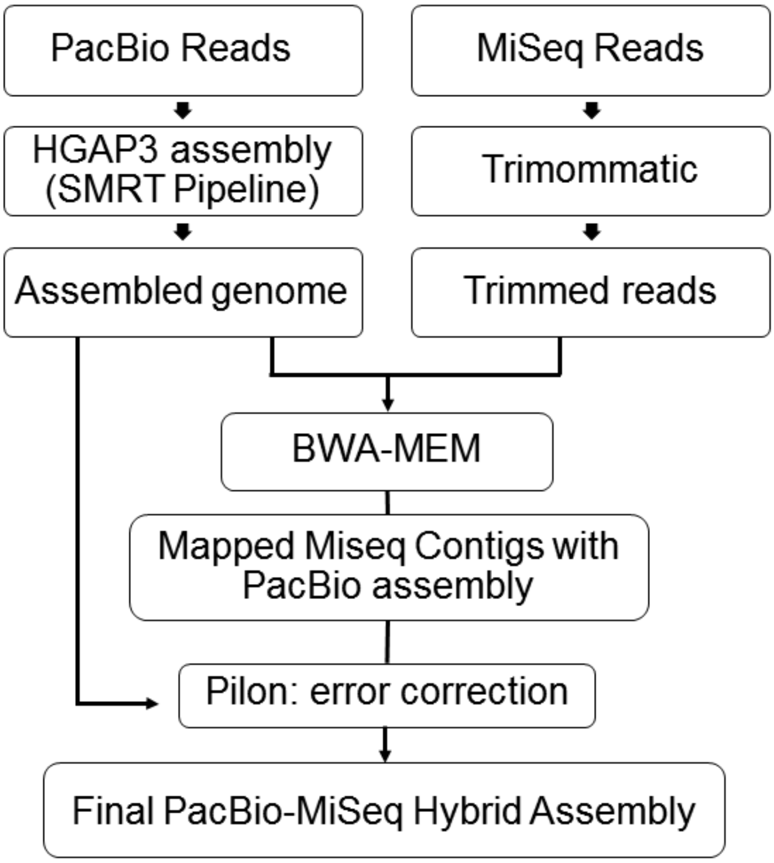

Figure S5. Flowchart depicting PacBio-Miseq hybrid genome assembly. The arrows indicate the sequential steps used for assembly.
