# Supplementary material for: Bacteriophages are the major drivers of Shigella flexneri serotype 1c genome plasticity: a complete genome analysis
Source: BMC Genomics. 2017 Sep 12;18:722. doi: 10.1186/s12864-017-4109-4 (PMC5596473; doi:10.1186/s12864-017-4109-4)
Supplement: Supplementary file 6 — Linear schematic representation of SHI-2 pathogenicity Island in Y394. (PDF 69 kb) [file 12864_2017_4109_MOESM6_ESM.pdf]

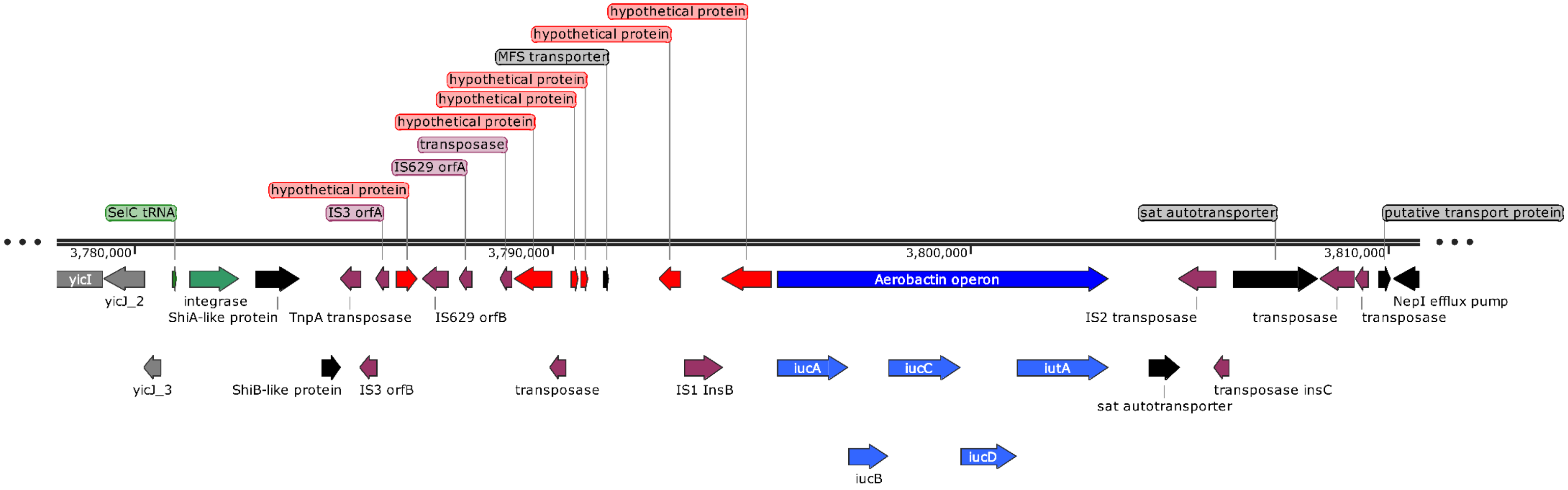

Figure S2. Linear schematic representation of SHI-2 pathogenicity Island in Y394. The direction of arrows indicates the orientation of open reading frames (ORFs). The color code denotes the ORF types: red, hypothetical protein; plum, transposase and IS elements, light green, tRNA; dark green, integrase; gray/black, annotated protein.
